# Letter from W. B. Huson, Sheboygan Falls, Wis.

**Published:** 1868-01-15

**Authors:** W. B. Huson

**Affiliations:** Cor. Sec’y.; Sheboygan Falls, Wis.


Sheboygan Falls, Wis., *December 20th*, 1867.

*Editor Chicago Medical Journal:*

I am requested by the Medical Association of this county, to inform you that a Medical Society was organized August

19th, 1867, entitled the Sheboygan County Medical Association. Its officers are as follows:

President, Dr. Louis Bock, of Sheboygan; Vice President, Dr. H. J. Young, of Sheboygan Falls; Recording Secretary, Almon Clarke, of Sheboygan Falls; Corresponding Secretary, W. B. Huson, of Sheboygan Falls; Treasurer, A. F. St.Sure, of Sheboygan. Censors, Henry Bodenstab, of Howard's Grove; J. N. O'Brien, of Plymouth; W. B. Huson, of Sheboygan Falls.

Its remaining members are: L. D. McIntosh, of Sheboygan; Fred. Hahn, of Sheboygan; W. D. Morehouse, of Plymouth; G. B. Shepard, of Sheboygan Falls; and J. J. Brown, of Sheboygan. Yours very truly,

W. B. Huson, Cor. Sec'y.

